# Research on spatial characteristics of metropolis development using nighttime light data: NTL based spatial characteristics of Beijing

**DOI:** 10.1371/journal.pone.0242663

**Published:** 2020-11-30

**Authors:** Yuli Yang, Mingguo Ma, Xiaobo Zhu, Wei Ge

**Affiliations:** 1 School of Civil Engineering, Lanzhou University of Technology, Lanzhou, China; 2 Emergency Mapping Engineering Research Center of Gansu, Lanzhou, Gansu, China; 3 Chongqing Engineering Research Center for Remote Sensing Big Data Application, Southwest University, Chongqing, China; 4 Chongqing Engineering Research Center for Spatial Big Data Intelligent Technology, Chongqing University of Posts and Telecommunications, Chongqing, China; 5 Shenzhen State High-Tech Industrial Innovation Center, Shenzhen, China; Northeastern University (Shenyang China), CHINA

## Abstract

As the capital and one of the metropolises in China, Beijing has met with a number of serious so-called "urban diseases" in the process of rapid urbanization such as blind expansion of urban areas, explosion of population and the increase of urban heat island effect. To treat these “urban diseases” and make the metropolis develop healthful and sustainable in Beijing in the future, the spatial characteristics of metropolis developments in Beijing are explored in this paper. The urban built-up areas in Beijing are extracted using the DMSP-OLS nighttime light data from 1992 to 2013. The characteristics of the urban developments of Beijing are studied, including spatial and temporal scales of urban developments, urban barycenter of Beijing and its transfer trajectory, variations of urban spatial forms and the differences of urban internal developments. The results have shown that the built-up areas had been increasing and circling extending from the central urban areas to the outer spaces in the last 21 years. The built-up area had expanded by 878km^2^ in 1992–2013, and the built-up area in 2013 had expanded to three times comparing to that of 1992. The expanding area of the built-up area in the northeast is the largest. The expansion of the urban had mainly occurred in 1996–2007, and the expanded area had accounted for 92% of the total research period. During the whole research period, the urban barycenter of Beijing had moved 5000.71 meters towards Northeast 28° of its original place from Dongcheng District to Chaoyang District. The development level of each municipal district had been increasing year by year, and the development differences among the municipal districts had been gradually reduced; the spatial forms of Beijing had been alternately changed between extensive and intensive expansion. The results of this study can help to plan urban land use and people migration of Beijing.

## 1. Introduction

China has experienced a rapid process of urbanization since its open-door policy. With the acceleration of urbanization, many "urban diseases", such as rapid increasing of urban population, disorder expansion of urban land use, reduction of cultivated land, environmental pollution, ecological deterioration and traffic congestion, have become more and more serious, and the resources and environments in many metropolises are difficult to support the development of the cities. In addition, rapid urbanization will lead to urban heat island effect, which negatively impact ecological and economic aspects of the cities, including urban atmospheric pollutant concentration, water consumption and higher health risks for residents. These affected the quality of life and the sustainable and healthy development of the cities. Therefore, it is of strategic significance to study and get the laws and characteristics of the spatio-temporal evolutions of the metropolises for determining their rational distributions of urban land and avoiding blind expansion of the cities, and to realize the sustainable developments of the regions [1].

As a type of new data sources, nighttime light data records night light brightness and may be used to monitor human nighttime activities. DMSP/OLS nighttime light images can characterize human activities such as population [2–5], economy [6–8] and urbanization levels [9–12]. It can reflect the intensity of human activities and urbanization development levels in various comprehensive factors. Indeed, it is a new and effective data source for monitoring the process of urbanization developments.

Most achievements on the characteristics of urban developments by DMSP/OLS data focus on the methods for extracting of urban built-up areas. Small et al. transformed global nighttime light images of three periods from 1992 to 2000 into observation frequency data, superposed the images with different colors, and directly judged the directions and trends of urban expansion by the colors [13]. Pandey et al. extracted the urban information of India from 1998 to 2008 using night lighting data and SPOT-VGT data, and evaluated the accuracy by comparing the Google Earth images, and obtained the two cities which had larger degree of urban expansion in coming ten years [14]. Small et al. monitored the cities in Asia from 1992 to 2009 by night light data, and revised the DN value of the data to improve the accuracy of urban spatial extraction [15]. Tan used a buffer model to extract 120 representative cities in North China in 2000 and validated them with Landsat images, and the results showed that the linear correlation between them is high [16]. He used nighttime light data and statistical data to reconstruct the urbanization process in China's mainland and Bohai Rim [17, 18]. Wang divided China into seven regions to extract large-scale urban land use information in China [19]. Yang [20] studied the spatial and temporal dynamics of land urbanization level in China. Guo [21] explored the expansion law of urban agglomerations in eight economic zones in China. The above achievements have contributed to the study of urbanization process. However, most of the research focused on the extraction of the urban built-up areas or urban expansion in large scale and the cities were not regarded as individuals with internal spatial heterogeneity to study their spatial development characteristics.

In this paper, DMSP/OLS nighttime light data is used as a comprehensive factor to characterize the intensity of human activities, and Beijing, the capital of China, is taken as an example of the metropolises that have been and are developing rapidly in urbanization. Beijing is obviously a space with internal differences to whose spatial characteristics of urban developments need to be studied and its development differences and the equilibrium degrees within the municipal districts is also worth investigating. So the following work has been done in our study: (1) the spatio-temporal scales of urban expansions are studied by analyzing the expansion speed, intensity and expansion types of the urban areas; (2) the urban barycenter in different periods of Beijing is extracted, and the transitional trajectory of urban barycenter is analyzed; (3) the compactness index is selected to analysis the change of the urban spatial forms; and (4) the coefficient of variation (CV) and relative development rate (ROR) are constructed by the total light amount in each jurisdiction to study the development differences in the city.

## 2. Materials and methods

### 2.1 Study area

**Fig 1:**
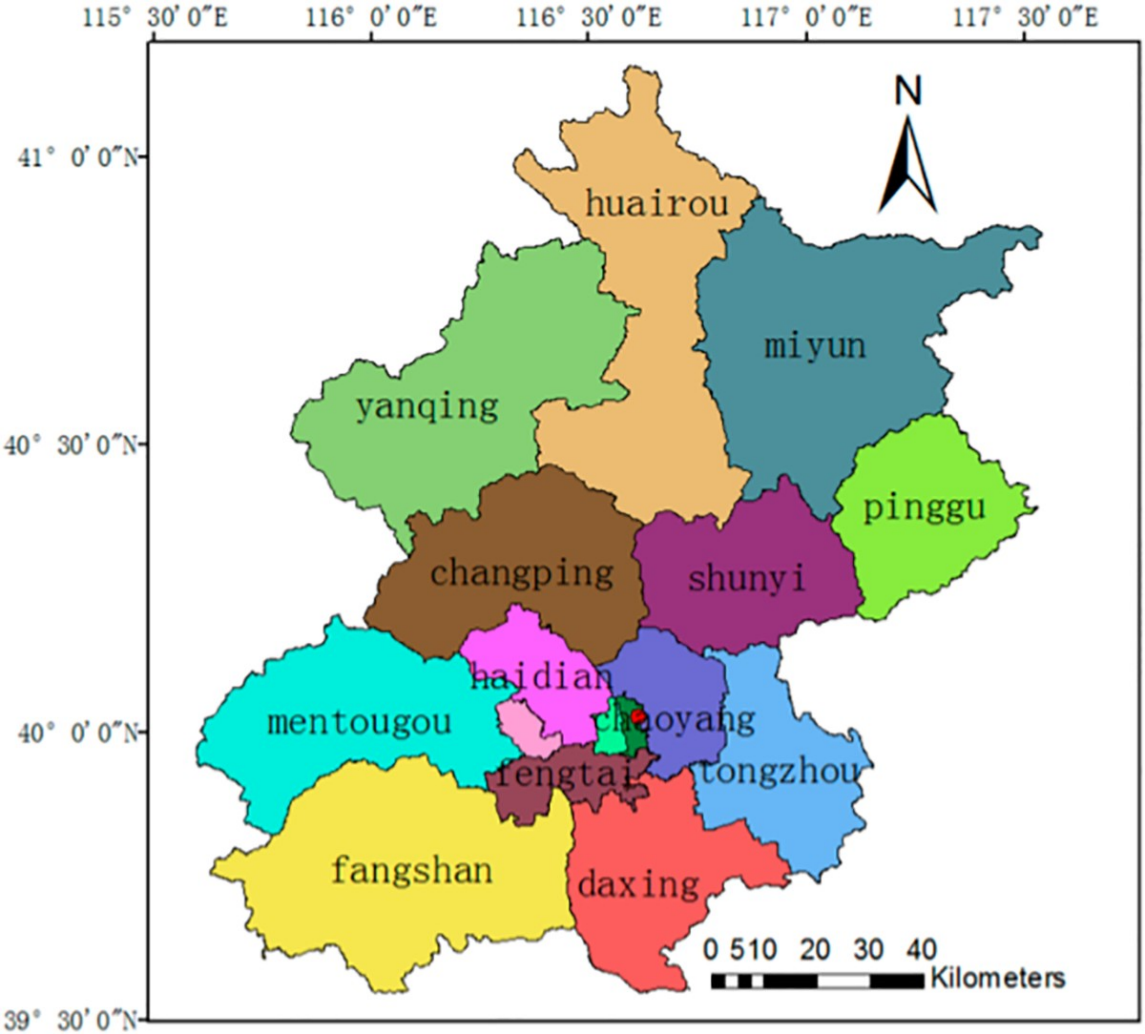
Administrative map of Beijing.

As one of the world’s largest cities, Beijing is the capital and the economic, political, cultural and international communication center of China. It has an important influence on the world and also has a large number of world cultural heritage sites. The center of Beijing is located at 39°54′ north and 116°23′ east, and the area of the city is 16,411 km^2^. The resident population of Beijing is 21,729,000 in 2016, and the population density is 1323 persons/ km2 in 2015 [22]. There are 16 districts in Beijing (Fig 1), including two core capital function zones: the Dongcheng District and Xicheng District; four city function expansion areas: the Chaoyang District, the Haidian District, the Shijingshan District, and the Fengtai District; five new city development districts: the Fangshan District, the Daxing District, the Tongzhou District, the Shunyi District, and the Changping District; and five ecological conservation development zones in Fig 1: the Mentougou District, the Yanqing District, the Huairou District, the Pinggu District, and the Miyun District [23].

After 40 years of rapid development since the reform and opening-up policy in China, Beijing has been facing tremendous pressures in population, resources, environment and society. Thus, it is of great significance to know its spatial development characteristics for the purpose of scientifically regulating its urbanization process.

### 2.2 Data source and data preprocessing

#### 2.2.1 Data source

The data used in this study includes the night-time light (NTL) data of DMSP-OLS from 1992 to 2013, administrative division data and statistical data in the corresponding years of Beijing. The version 4 DMSP-OLS nighttime light time data is collected from the website of NOAA’s National Centers for Environmental Information (NCEI, formerly NGDC) [24]. The statistical data of Beijing is obtained from the Beijing Statistical Yearbook, an annual statistical publication compiled by the National Bureau of Statistics, China, containing economic and social statistics of past years. The administrative boundaries of Beijing, including all districts, are collected from the National Geomatics Center of China.

The Defense Meteorological Satellite Program (DMSP) of the United States is equipped with an Operational Linescan System (OLS) sensor, which can work at night and detect light from cities or even low-intensity light from small-scale residential areas and vehicles. Lights make city areas obviously different from dark country backgrounds [25] and are more suitable for the dynamic monitoring of urbanization. There are three types of annually averaged data in the dataset: cloud free coverage, average visible light, and stable lights. Among the three types of data, the stable light data contain the lights derived from cities, towns, and other sites with persistent lighting, while fires, volcanoes, background noise, and other ephemeral events have been discarded [26]. The DMSP-OLS nighttime stable light (NSL) data have a spatial resolution of 30 arc-seconds, about 1 km at the equator, and a coverage spanning −180° to 180° and −65°S to 75°N. The digital number (DN) values of pixels range from 0 to 63. A value of 0 represents an unlit area and the greater the value, the higher the light level of the region. In addition, the NOAA/NCEI website has released a global radiance calibrated nighttime light (RCNL) dataset without sensor saturation, which can be used as the ideal reference data for the intercalibration of the DMSP-OLS NSL dataset [27–31].

#### 2.2.2 Data preprocessing

In this study, the DMSP-OLS NSL data in 1992–2013 is used, and it is preprocessed and corrected before use. The original data in the WGS84 reference coordinate system is changed to the Chinese Lambert conformal conic projection using ArcGIS 10.3 (ESRI, Redland, CA, USA). The central meridian of the projection is at 105° E, and the two standard parallels are 30° N and 62° N. DMSP-OLS series data are acquired by different satellites in different years using different sensors, and the data in the same year is also composed of multiple satellite image data. The data has no on-board calibration and saturation of bright lights. Therefore, it is necessary to correct the DMSP-OLS data to improve its continuity and accuracy [32, 33].

Firstly, the DMSP-OLS data is intercalibrated. The DMSP-OLS data from 1992 to 2013 is intercalibrated by the invariant region method [30, 34]. According to the work of Meng et al. [34], Japan is selected as an invariant region. Because Japan had experienced a relatively stable socioeconomic development and had a wide spread of DN values within the NSL data, these characteristics can improve the accuracy of intercalibration; and the 2006 RCNL data is selected as the reference image [30]. Then, the DMSP-OLS data from 1992 to 2013 is intercalibrated using the power function regression model [35, 36].

Secondly, the data is intra-annually composited. In a certain year, the DMSP-OLS data is collected from two satellites simultaneously. To improve its accuracy, the DN values of the images from multiple satellites are averaged. This is called the intra-annual composite of DMSP-OLS data.

**Fig 2:**
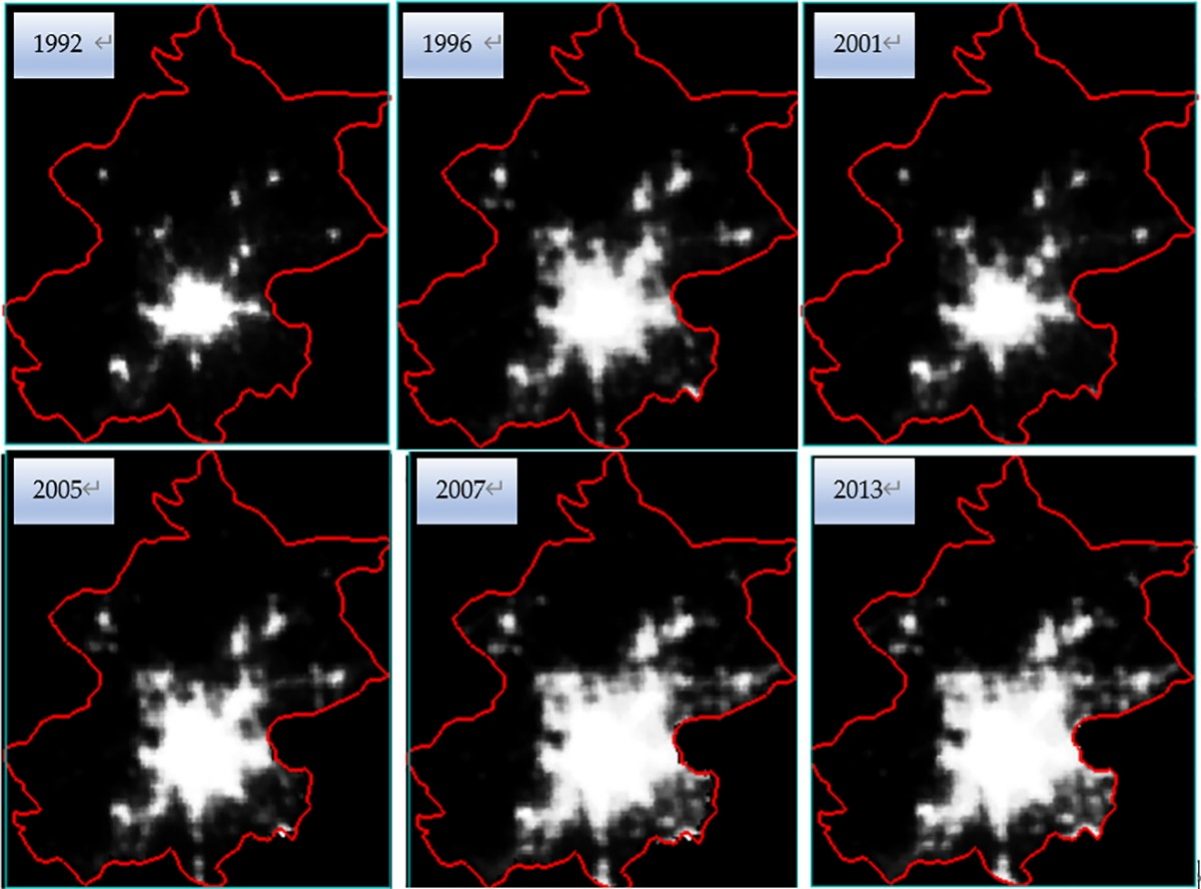
Corrected DMSP/OLS night-time light images of Beijing.

Thirdly, the inter-annual series correction is done. It is assumed that the DN value of each pixel detected in a given year should not be less than that detected for the same pixel in the previous year [33]. According to this principle, the DMSP-OLS data from 1992 to 2013 is corrected. After the correction, the NSL data of Beijing can be obtained by clipping the DMSP-OLS NSL data in 1992–2013 using the administrative boundary of Beijing. The corrected DMSP-OLS image of Beijing in six periods from 1992–2013 are shown in Fig 2.

### 2.3 Method

#### 2.3.1 Extraction of urban built-up areas of Beijing

The threshold dichotomy comparison method based on statistical data [13] is improved to get the boundaries of the build-up areas more accurately. The procedure of the improved method is as follows:

Firstly, the area values of the built-up areas of Beijing over the past years is collected from the statistical yearbook. The night stable light data of Beijing in 1992, 1996, 2001, 2007 and 2013 in five periods is selected and pre-processed to extract the built-up areas.

**Fig 3:**
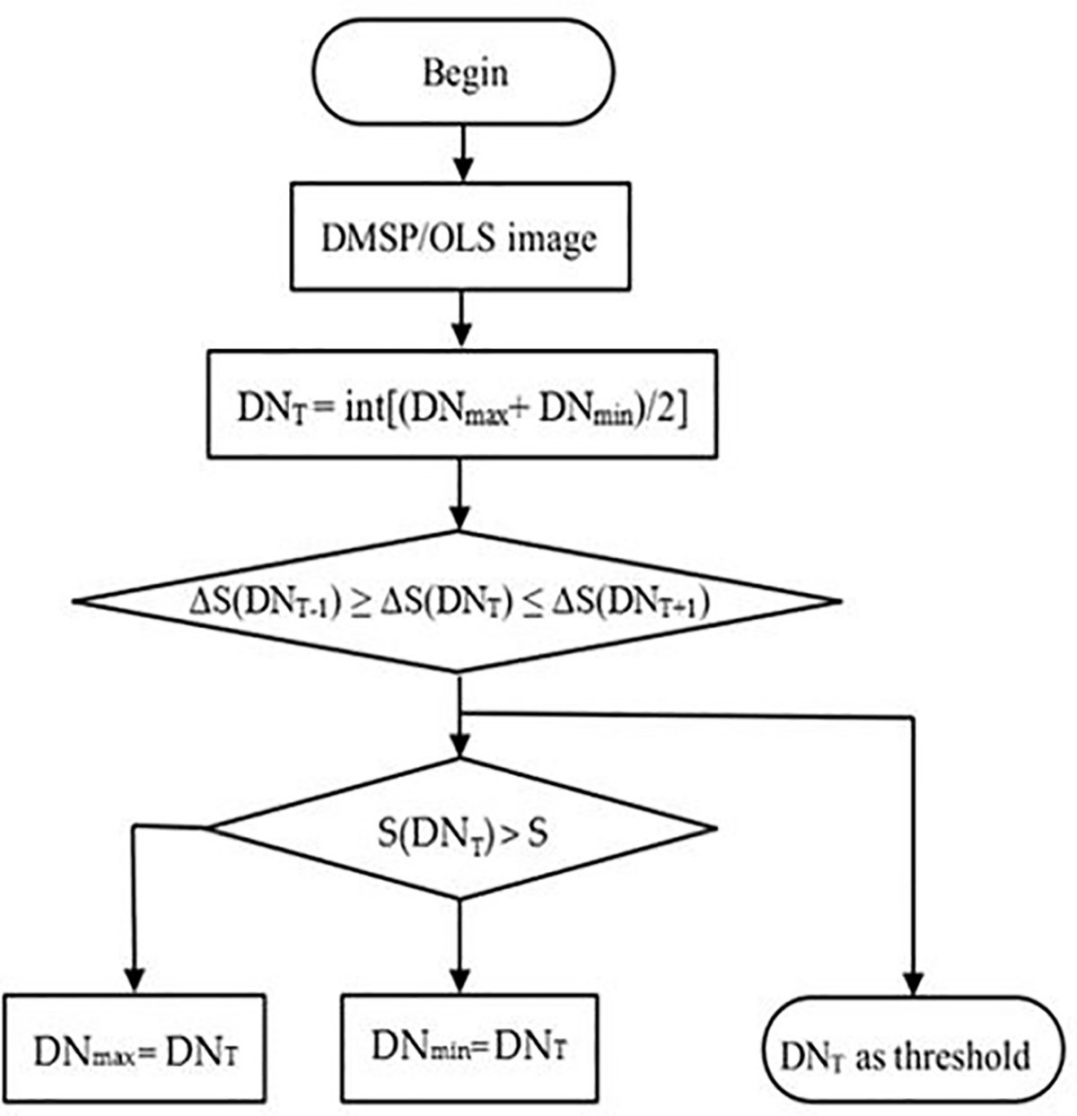
Procedure of 1^st^ phase for the light data extraction.

Secondly, the threshold of the built-up area of Beijing in the first year (1992) is set, and the area of the build-up area under the threshold is calculated (See Fig 3).

Supposing that the maximum and minimum DN values of the NSL data in 1992 are DN_max_ and DN_min_, respectively, and the area of the built-up area in the same year in the statistical yearbook is S, and the initial extraction threshold DN_T_ of the built-up area in Beijing is set by formula (1).

$${\mathrm{DN}}_{T}=\mathrm{int}\left[\left({\mathrm{DN}}_{\max}+{\mathrm{DN}}_{\min}\right)/2\right]$$ (1)

The extracted area of light patches in the study area is S(DN_T_). ΔS(DN_T_) is the absolute value of the difference between S(DN_T_) and statistical data S. The threshold is adjusted continuously by dichotomy to make the value of ΔS(DN_T_) to a minimum, and this threshold is just the finally extracted threshold.

**Fig 4:**
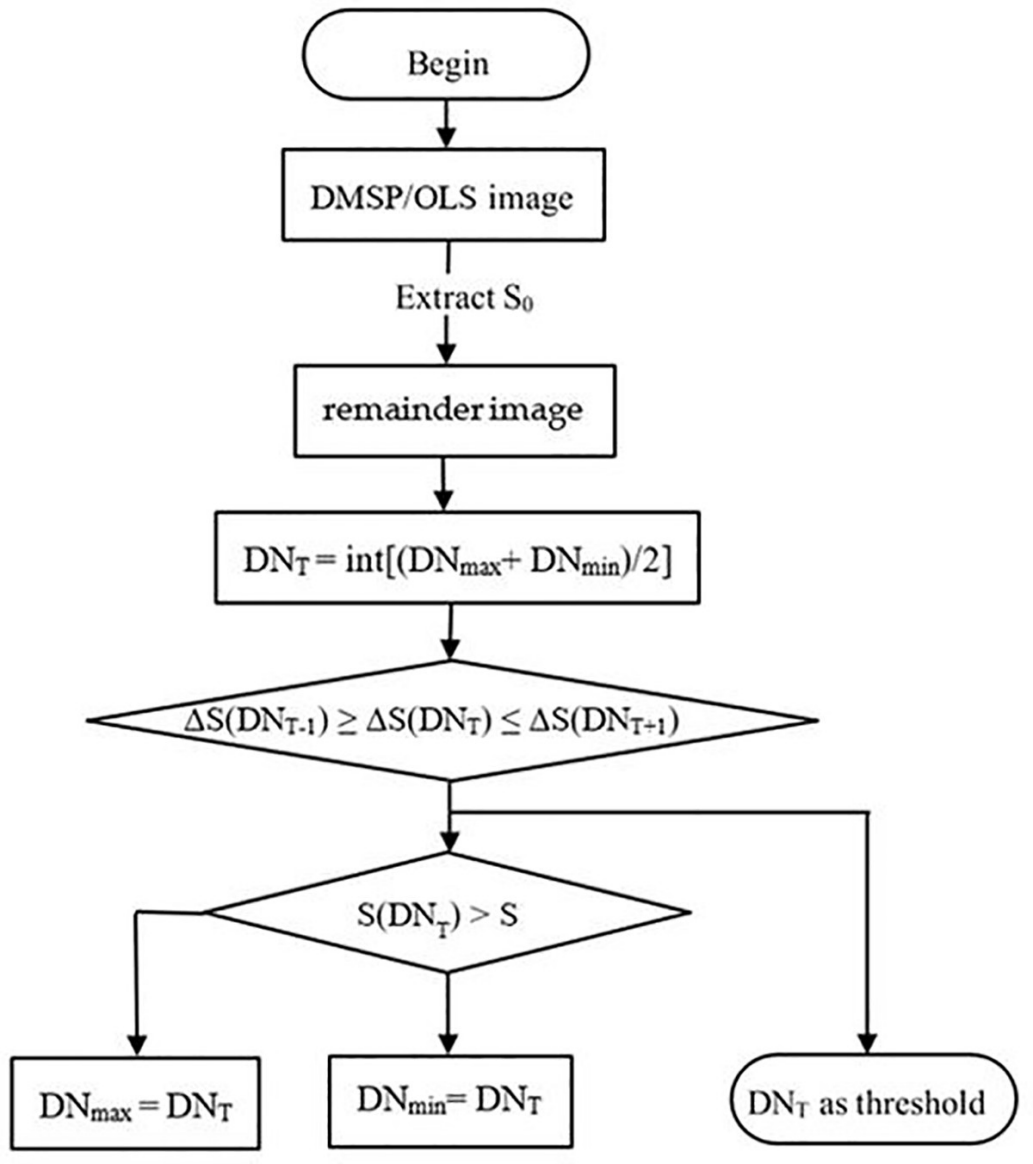
Procedure of light data extraction using previous results.

Thirdly, after extracting the built-up area of 1992, the same method is used to extract the built-up areas of the other periods. In a subsequent period, the image of built-up area in the previous period which has been extracted is preserved, and the patch area is S_0_. S (DN_T_) is the newly extracted patch area. ΔS(DN_T_) is the absolute value of the difference between S(DN_T_) + S_0_ and statistical data S. Then the new threshold is set to extract the built-up area in the subsequent period. This procedure is shown in Fig 4.

After the DMSP-OLS data is intercalibrated, the range of the DN values is stretched from 0–63 to 0–255. The thresholds and the extracted built-up areas in different periods are shown in Table 1.

**Table 1:** Areas of the built-up area in different periods.

Year	Extraction area (km^2^)	Statistical data (km^2^)	Threshold	Relative error (%)
**1992**	436	429.4	225.5	1.54
**1996**	483	476.8	219.5	1.30
**2001**	751	747.77	213.3	0.43
**2007**	1290	1289.32	220.3	0.05
**2013**	1314	1306.5	216.9	0.57

#### 2.3.2 Spatio-temporal scales of urban development

The spatio-temporal scales of urban development of Beijing are studied by expansion speed, expansion intensity and expansion type index.

- Expansion speed

Expansion speed refers to the annual growth rate of urban land use, which indicates the speed of urban land expansion. It may be calculated by:

$$V={\left(S_{m}‐S_{c}\right)}_{}/T$$ (2)

where, V is the expansion speed; S_c_ and S_m_ are the area of the built-up area in the initial study period and that in the finale study period, respectively; and T is the time interval.

- Expansion intensity

Expansion intensity is the expansion extent of urban land area in unit time, which indicates the strength or weakness of urban land expansion [37]. It may be calculated by:

$$E={\left(S_{m}‐S_{c}\right)}_{}/\left(S_{c}\times T\right)$$ (3)

where, E is the expansion intensity of the city.

- Expansion type index

Expansion type index refers to the ratio of the expansion speed of urban land in a certain period to that of the previous period [38]. It may be calculated by:

$$U=V_{t2‐t3}/V_{t1‐t2}$$ (4)

where, U is the Expansion type index; V_t2-t3_ is the expansion speed in the period t2-t3; and V_t1-t2_ is the expansion speed in the period t1-t2. If U>1, it is an accelerated expansion; if 0<U<1, it is a decelerated expansion; and if U = 1, it is a uniform expansion.

#### 2.3.3 Barycenter and direction of urban development

The barycenter of a city can be regarded as the average position of the city, which is an important measure for describing the spatial distribution of the city, as well as a balance point for maintaining a uniform distribution of the city [39]. Therefore, the change of urban spatial expansion can be reflected by the change of urban distribution barycenter.

The patches of built-up areas extracted from night light data can be used as a symbols of human activity. To calculate the barycenter of the extracted patches of built-up areas, the area of each patch is viewed as the weight, and the barycenters of the built-up areas patches of each period are extracted, and the coordinates of the barycenters can be calculated. If the coordinates are calculated in ArcGIS 10.3 using Mean Center tool under Spatial Statistics Tools in ArctoolBox, the formula are as follows:

$$\begin{array}{l} X_{t}=\sum_{i=1}^{n}\left(C_{ti}\times X_{i}\right)/\sum_{i=1}^{n}C_{ti}\\ Y_{t}=\sum_{i=1}^{n}\left(C_{ti}\times Y_{i}\right)/\sum_{i=1}^{n}C_{ti} \end{array}$$ (5)

where, X_t_ and Y_t_ are the barycenter coordinates of the built-up area in Beijing in year t; C_ti_ is the area of the ith patch in year t; X_i_ and Y_i_ are the coordinates of the geometric center of the ith patches.

Here, the city barycenters in 1992, 1996, 2001, 2005, 2009 and 2015 are extracted. The transfer trajectory of the city barycenters may be studied using the following three indexes: transfer distance, transfer speed and transfer angle.

The transfer distance is the average distance that the city barycenter moves in a certain period, shown in Formula (6).

$$D_{t=}\sqrt{{\left(X_{t}-X_{t-1}\right)}^{2}+{\left(Y_{t}-Y_{t-1}\right)}^{2}}$$ (6)

Transfer speed is the average speed of the city barycenter moving in a certain period, shown in Formula (7). T is the time interval.

$$V_{t}=D_{t}/T$$ (7)

Taking the oriental direction as the starting reference direction, the angle between the moving direction of the city barycenter to the staring reference direction is called the transfer angle. The transfer angle can be calculated by Formula (8).

$$\theta_{t}=n\pi +\arctan\left(\frac{Y_{t}-Y_{t-1}}{X_{t}-X_{t-1}}\right)\;(n=0,1,2)$$ (8)

where, X_t_, Y_t_ and X_t-1_, Y_t-1_ are the coordinates of the city barycenter in year t and t-1, respectively.

#### 2.3.4 Spatial forms of urban development

Urban expansion can lead to the change of urban spatial form. The urban spatial form can be reflected by the compactness of the peripheral outline form of the built-up areas. The expression of compactness index is as follows:

BCI=2πAP (9)

where, BCI is the compactness index of the urban built-up areas; P is the perimeter of the outline of the built-up areas; and A is the area of the urban built-up area. In a certain period, decrease of the compactness indicates that the urban extension expansion is dominant; while increase of the compactness indicates that the filling intensive expansion is dominant in this period.

#### 2.3.5 Equilibrium degree of urban internal development

The difference of urban internal development of Beijing is reflected by the difference of the total amount of lighting in different municipalities. In order to facilitate the statistics of the total amount of lighting in the municipal districts, the corrected night lighting data of each year in Beijing are normalized by Formula (10) to make the gray value of the pixels in the range of 0–1.

$${\mathrm{DN}}_{g}=\frac{{\mathrm{DN}}_{i}-{\mathrm{DN}}_{\min}}{{\mathrm{DN}}_{\max}-{\mathrm{DN}}_{\min}}$$ (10)

where, DN_g_ is the gray value of the raster pixel after normalization; DN_i_ is the gray value of the i^th^ grade raster pixel; and DN_max_ and DN_min_ are the maximum and the minimum gray values of the raster pixel in the study area, respectively.

The normalized light brightness value is used to construct the index to study the urban internal development equilibrium, and it is studied by the difference of urban internal development and the relatively development rate of Beijing.

*2*.*3*.*5*.*1 Differences of urban internal development*. As far as the sixteen municipal districts of Beijing are concerned, the comprehensive index of regional development level is represented by the total sum of regional light (SL), and the overall level of urban internal development difference is calculated by standard deviation (SD) and coefficient of variation (CV) [40]. The calculation formulas are as follows:

$$\mathrm{SL}=\sum_{i}{\mathrm{DN}}_{i}\times C_{i}$$ (11)

$$\mathrm{SD}=\sqrt{\frac{\sum_{i=1}^{n}{\left(SL_{i}-SL_{p}\right)}^{2}}{n}}$$ (12)

$$\mathrm{CV}=\frac{\mathrm{SD}}{\mathrm{SL}}$$ (13)

In Formula (11), SL is the total amount of regional lighting; DN_i_ is the gray value of the i^th^ grade; and C_i_ is the grid number of the i^th^ grade gray. In Formula (12), SD is the standard deviation; n is the number of municipal districts; SL_i_ is the total amount of lighting of the i^th^ municipal district; and SL_p_ is the average value of the lighting of each municipal district. In Formula (13), CV is the coefficient of variation. In different research periods, increase of the value of CV indicates that the gap of development level of different municipal districts is increasing, while decreases of the value of CV indicates that the gap of development level of different municipal districts is decreasing.

*2*.*3*.*5*.*2 Relative development rate of municipal districts in the urban*. The relative development rate (RDR) is used to measure the spatial characteristics of the economic development rate of each municipal district in a city. RDR indicates the ratio between the change of the development level of a municipal district and the change of the whole urban development level in a certain period [41]. It can be defined by Formula (14):

$$\mathrm{RDR}=\frac{{\mathrm{SL}}_{2i}-{\mathrm{SL}}_{1i}}{{\mathrm{SL}}_{2}-{\mathrm{SL}}_{1}}$$ (14)

where, SL_2i_ and S1_1i_ are the total amount of lighting at the end and early stages of the study period, respectively, in the i^th^ municipal district; and SL_2_ and SL_1_ are the total amount of lighting at the end and early stages of the study period, respectively, in the whole city. A greater RDR value indicates that the relative development rate of the municipal districts is higher, while a less RDR value indicates that the relative development rate of the municipal districts is lower in this period.

## 3. Results

### 3.1 Temporal and spatial scales analysis of urban expansion

**Fig 5:**
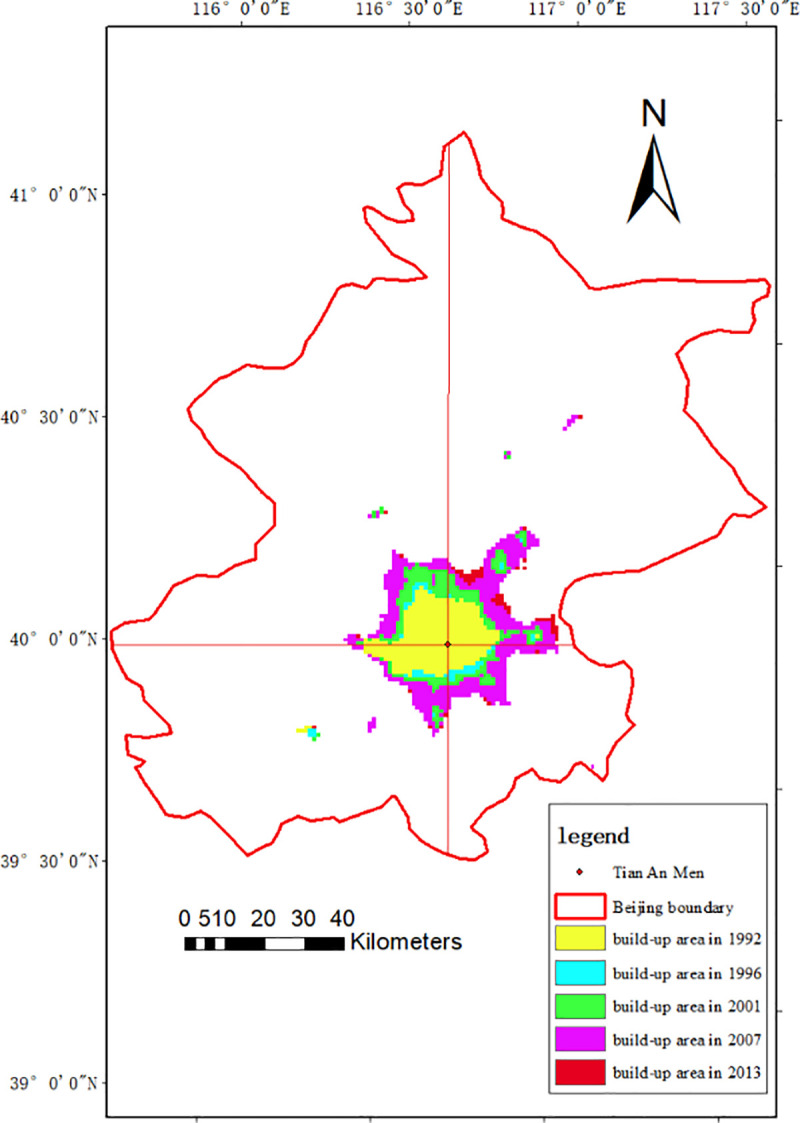
Expansion of the built-up area in four quadrants of Beijing in 1992–2013.

Taking the five periods of 1992, 1996, 2001, 2007 and 2013 as the dividing point, the built-up area of each period is extracted and the expansion of the built-up area at each stage of Beijing is analyzed. The built-up area in each period are displayed in different colors. The overlay map of the built-up areas from 1992 to 2013 in Beijing is shown in Fig 5.

From Fig 5, it can be concluded that the built-up area of Beijing from 1992 to 2013 expanded from the city center to the outer ring.

The area of the built-up area in the five periods are known. The expansion speed, intensity and the expansion type index of Beijing in each stage are calculated. The results are also listed in Table 2.

**Table 2:** Expansion of the built-up areas at each stage in Beijing.

Research period	Time	Extending area(km^2^)	Extending speed(km^2^/a)	Expansion Intensity (%)	Contribution rate (%)	Expansion Type Index(U)
**First stage**	1992–1996	47	11.75	2.69	5.35	
**Second stage**	1996–2001	268	53.6	11.1	30.52	4.56
**Third stage phase**	2001–2007	539	89.833	11.96	61.39	1.68
**Fourth stage**	2007–2013	24	3.429	0.27	2.73	0.04
**Total stage**	1992–2013	878	41.81	9.59	100	

From Table 2, it can be concluded that among the four stages, the highest expansion speed, the greatest intensity and the greatest contribution rate of the built-up area are in the third stage in 2001–2007, and the second stage in 1996–2001 takes the second place, and the fourth stage in 2007–2013 is the lowest. The urban expansion areas in the second and third stages accounted for 92% of the total research period and only 2.89% in the fourth stage. Therefore, the urban expansion of Beijing is mainly concentrated in 1996–2007. In 1996–2007, the expansion type index U>1, so they belongs to accelerated expansion in the second and third stage; In 2007–2013, 0<U<1, so it is a decelerated expansion in the fourth stage.

**Fig 6:**
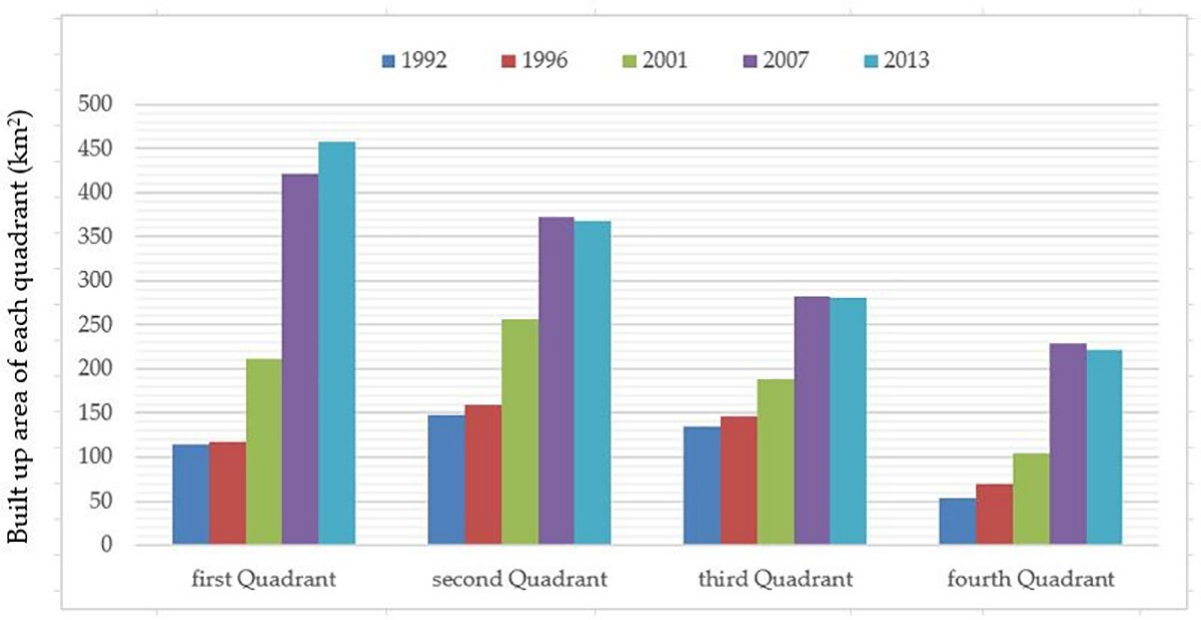
Built up areas of the four quadrants in Beijing in each period.

To study the expansion of the built-up area in different directions, the coordinate system is established with Tian An Men as the origin of coordinates and the east direction as the increasing direction of horizontal axis. The plane is divided into four quadrants (Fig 5). The areas of the built-up areas in four quadrants in Beijing in 1992 and 2013 is counted and drawed using a column chart (Fig 6).

From Fig 6, it can be concluded that the areas of four quadrants in Beijing are all expanding outwards in the whole study period. The first and second quadrants have larger expansion areas, and the third and fourth quadrants have smaller expansion areas, and the first quadrant has the largest expansion area. Therefore, the area of Beijing expanding northward is larger than that of south, and the expanding area towards northeast is the largest.

### 3.2 Analysis of the city barycenter transfer trajectory and development direction

Firstly, the building up areas in 1992, 1996, 2001, 2005, 2009 and 2013 are extracted, respectively. Then, using ArcGIS 10.3, the city barycenters in the corresponding six periods are obtained by the "mean center" operation taking areas as weights.

**Fig 7:**
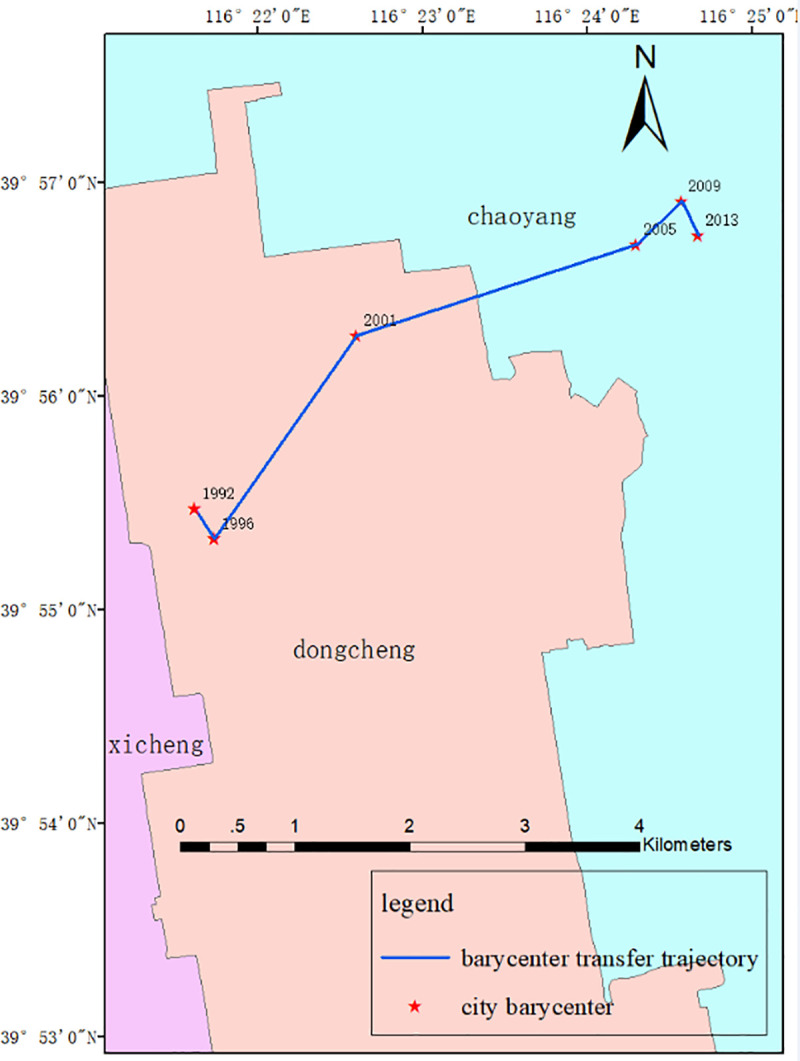
City barycenter transfer trajectory in Beijing.

The transfer characteristics of the city barycenter of Beijing are analyzed (Fig 7). Here, the distance, speed and angle of the urban center of gravity transfer in each period are calculated (shown in Table 3). The starting direction is the oriental direction, and the counter-clockwise rotation is the positive direction.

**Table 3:** Barycenter transfer characteristic of Beijing.

Feature parameters	First stage	Second stage	Third stage	Fourth stage	Fifth stage	Total stage
	(1992–1996)	(1996–2001)	(2001–2005)	(2005–2009)	(2009–2013)	(1992–2013)
**Transfer distance (m)**	314.69	1688.91	2562.89	544.86	334.05	5000.71
**Transfer speed (m/a)**	78.67	337.78	640.72	138.72	85.51	227.305
**Transfer angle (°)**	East by south 57°	East by north 58°	East by north 19°	East by north 45°	East by south 63°	East by north 28°

From Fig 7, it can be concluded that from 1992 to 2013, the city barycenter of Beijing has been gradually moving eastward. From Fig 7 and Table 3, it can be concluded that the largest moving distance of the city barycenter is in the third stage from 2001 to 2005, and then are the second stage, the first stage and the fifth stage. It is obvious that the moving speed of the city barycenter at each stage has the same order as that of the moving distance.

The city barycenter of Beijing was located in Dongcheng District in 1992, and its geographical coordinates was 116° 21′ 45″ E, 39° 55′ 30″ N. In the first stage, the city barycenter moved to 57° east by south, 58° east by north in the second stage, 19° east by north in the third stage, 45° east by north in the fourth stage, 63° east by south in the fifth stage. The city barycenter shifted to Chaoyang District in 2013 and its geographic coordinates were 116° 24′ 40″ E and 39° 56′ 30″ N. During the whole research period in 1992 to 2013, the shifting distance of the city barycenter was 5000.71m, and the city barycenter moved to 28° east by south with an average speed of 227.3 m each year.

### 3.3 Space morphological analysis of urban development

There is a direct causal relationship between the change of urban spatial forms and the development and expansion of the city. The compactness of the outline forms of the built-up areas can reflect the spatial forms of urban land. The compactness index is selected to evaluate it quantitatively. The compactness indexes of Beijing in six periods from 1992 to 2013 are calculated according to its definition formula. The results are shown in Table 4.

**Table 4:** Compactness indexes of the built-up areas in Beijing in 1992–2013.

Year	Area(km^2^)	perimeter(km)	compactness index (BCI)
**1992**	436	126	0.583
**1996**	483	128	0.598
**2001**	751	137	0.674
**2005**	1186	274	0.446
**2009**	1316	302	0.426
**2013**	1320	304	0.457

**Fig 8:**
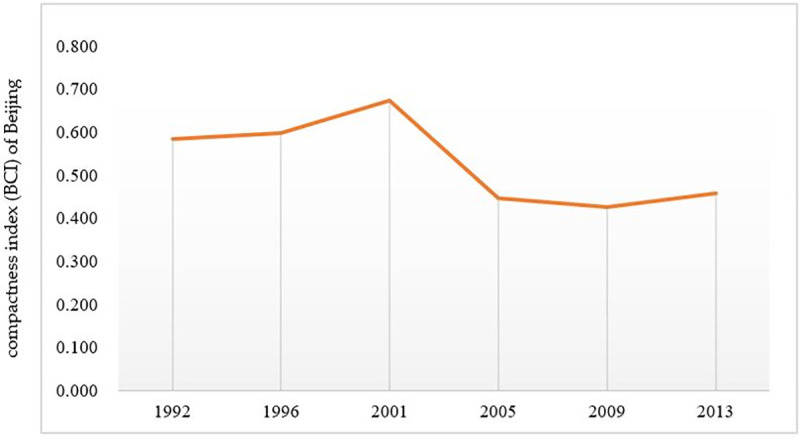
Variation trend of compactness index in Beijing during 1992–2013.

The compactness index change is shown in Fig 8. It can be concluded that the compactness index of Beijing had been gradually rising from 1992 to 2001, indicating that urban expansion in this stage is mainly belongs to connotative filling expansion type; while urban compactness index had been decreasing from 2001 to 2009, indicating that urban expansion in this stage is mainly belongs to extensive expansion type. And the compactness index of the city increased slightly from 2009 to 2013, showing that the urban expansion is mainly connotative filling development in this period. During the whole research period, the expansion type of Beijing had changed alternately between connotative and extension expansion.

### 3.4 Analysis of the equilibrium degree of urban internal development

#### 3.4.1 Analysis of the Coefficient of Variability (CV) of the city

To study the internal development differences among the municipal districts of Beijing, the sum of light (SL) in each municipal district is taken as a comprehensive index to characterize the development level of the municipal district, and the overall level of urban internal development differences is measured by the standard deviation (SD) and the coefficient of variation (CV).

The corrected DMSP-OLS night-time light data in 1992, 1996, 2001, 2007 and 2013 of Beijing are normalized by Formula (10). Then they are clipped by the boundary of Beijing administrative region, and the night-time light data of 16 municipal districts of Beijing in each period can be obtained. In ENVI 5.3, SL in Beijing and each municipal district can be got by the “compute statistic” operation. SL, SD and CV can be calculated by Formula (11), Formula (12) and Formula (13). The results are shown in Table 5.

**Table 5:** The total DN values of light and variation coefficient of municipal districts of Beijing in 1992–2013.

District	Sum of the light in each district (SL)				
	1992	1996	2001	2007	2013
**Dongcheng**	43.82	43.8	43.71	43.83	43.83
**Xicheng**	49.72	49.82	49.69	49.82	49.82
**Chaoyang**	339.85	380.15	420.51	447.45	450.27
**Fengtai**	98.17	139.95	158.01	201.54	237.36
**Shijingshan**	45.08	54.88	58.12	70.47	77.77
**Haidian**	156.53	167.85	208.89	250.62	294.49
**Fangshan**	80.96	127.83	140.72	178.88	282.54
**Tongzhou**	75.05	135.99	216.67	348.14	519.81
**Shunyi**	74.82	162.96	253.63	399.25	582.15
**Changing**	94.3	172.72	273.05	358.59	491.81
**Daxing**	47.12	90.76	127.43	234.65	362.87
**Mentougou**	8.18	10.53	12.12	23.11	46.82
**Huairou**	20.12	38.79	60.96	85.55	131.3
**Pinggu**	17.22	31.39	47.81	74.1	122.99
**Miyun**	22.25	35.98	66.56	94.58	150.52
**Yanqing**	9.45	20.88	43.87	47.17	72.18
**Average value of SL**	73.915	109.56	136.36	170.64	244.78
**Total DN of Beijing(DN)**	1182.64	1643.4	2181.75	2881.78	3916.53
**standard deviation(SD)**	78.62	90.55	108.99	135.60	180.70
**Coefficient of variation (CV)**	0.0665	0.0551	0.0500	0.0471	0.04614

**Fig 9:**
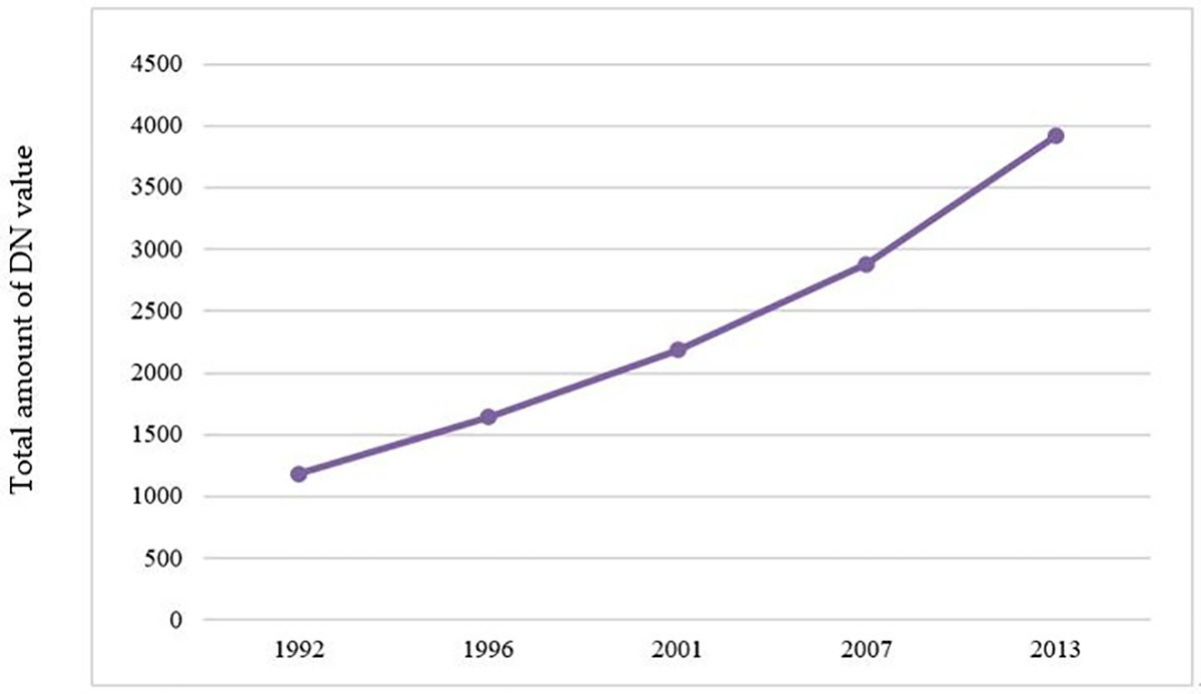
Total amount of DN value in Beijing.

**Fig 10:**
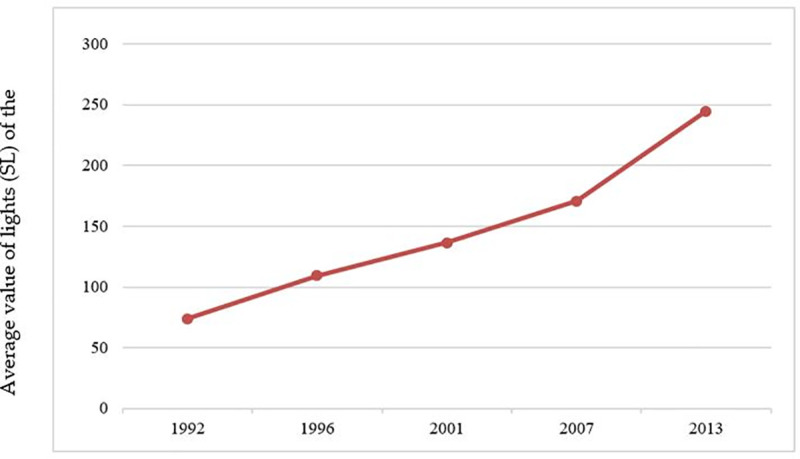
Average value of total lights (SL) of municipal districts in Beijing.

**Fig 11:**
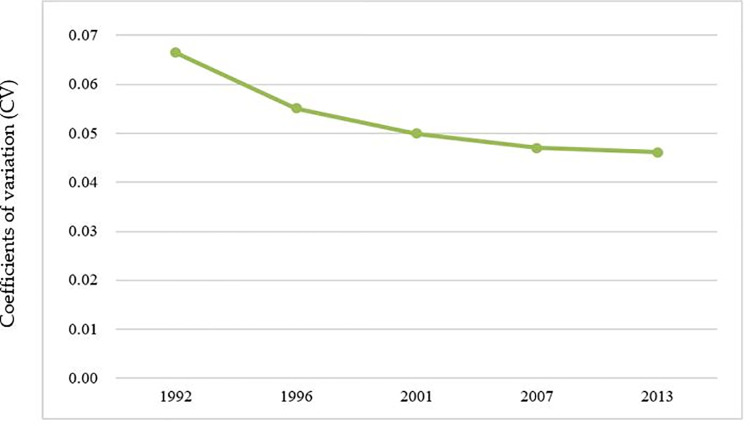
Coefficients of variation (CV) of urban development in Beijing.

The total amount of DN in Beijing and the average SL of the municipal districts in Beijing in the five periods are shown in Figs 9 and 10. The CV and its change trend in each period are shown in Fig 11.

From Fig 9, it can be concluded that the total light intensity of Beijing had also been increasing, and the overall level of urban development had been gradually improving. Fig 10 shows that the average light value of municipal districts gradually increases from 1992 to 2013. Fig 11 tells that the coefficient of variation (CV) decreases gradually from 1992 to 2013, which indicates that the gap of development level among municipal districts had been gradually decreasing.

#### 3.4.2. Analysis of the relative development rate of the city

The spatial characteristics of the economic development rate of the municipal districts in Beijing are measured by the index of relative development rate. According to the total light amount of each municipal district in Table 5 and the definition of the relative development rate (RDR) in each period, the RDR of each period can be calculated by Formula (14) and the results are listed in Table 6.

**Table 6:** The relative development rate of municipal districts of Beijing in each stage (RDR).

District	First phase	Second phase	Third phase	Fourth phase	Total phase
	1992–1996	1996–2001	2001–2007	2007–2013	1992–2013
**Dongcheng**	0.000043	0.000167	0.000171	0.000000	0.000004
**Xicheng**	0.000217	0.000241	0.000186	0.000000	0.000037
**Chaoyang**	0.087464	0.074970	0.038484	0.002725	0.040389
**Fengtai**	0.090676	0.033547	0.062183	0.034617	0.050913
**Shijingshan**	0.021269	0.006018	0.017642	0.007055	0.011957
**Haidian**	0.024568	0.076233	0.059612	0.042397	0.050463
**Fangshan**	0.101723	0.023944	0.054512	0.100179	0.073734
**Tongzhou**	0.132260	0.149865	0.187734	0.165905	0.162684
**Shunyi**	0.191293	0.168422	0.208020	0.176758	0.185571
**Changing**	0.170197	0.186366	0.122195	0.128746	0.145401
**Daxing**	0.094713	0.068116	0.153165	0.123914	0.115495
**Mentougou**	0.005100	0.002953	0.015699	0.022914	0.014134
**Huairou**	0.040520	0.041181	0.035127	0.044214	0.040667
**Pinggu**	0.030754	0.030501	0.037556	0.047248	0.038688
**Miyun**	0.029799	0.056803	0.040027	0.054061	0.046918
**Yanqing**	0.024807	0.042705	0.004714	0.024170	0.022945

Table 6 manifests that the RDR of Dongcheng District and that of Xicheng District in Beijing are the two lowest ones among all RDRs in all research periods, and the RDR in the fourth research stage and the whole research period is close to 0. It shows that as the core functional areas of the capital and the old urban areas in the center of Beijing, Dongcheng District and Xicheng District have been developed in saturation in the early stage. Thus, the urban areas gradually extend outward taking the old urban area as the developing center.

The RDR of Chaoyang District and Haidian District had been decreasing from the second stage to the fourth stage, which indicates that the development speed of the two districts, as new urban development functional areas close to the central jurisdiction, had been gradually slowing down and becoming saturated.

During the whole research period from 1992 to 2013, Fangshan, Tongzhou, Shunyi, Changping and Daxing districts, the five municipal districts as new urban development zone, had the highest relative development rate in each research period. Because the five districts belong to the new urban development zone of Beijing and the government had invested a great amount of money in them, and they had been in the period of quick development and prosperity. The RDR of Haidian District, Fengtai District, Miyun District, Huairou District and Pinggu District take the second place.

## 4. Discussion

It has some advantages to study the spatial characteristics of urban development using night-time light data, e.g. it can reflect urban internal development differences and the spatial characteristics of urban internal development rates, and it can break the limitation of previous work that treats urban built-up areas as a homogeneous space and unable to reflect internal development differences of the city.

However, there are also deficiencies in this study. The resolution of DMSP-OLS night-time light data is not very high, and there is light overflow or oversaturation in the urban central areas. Hence, it is necessary to further study on extracting built-up areas combining high resolution remote sensing data. In addition, in this study, only DMSP-OLS night-time light data is used. Cities in the future are always changing; thus, it is also necessary to study the spatial and temporal distribution and dynamic evolution characteristics of the cities using some newer and higher resolution night-time light data such as the data from NPP-VIIRS and Luojia-1 in the future.

The NTL data of DMSP-OLS used in this paper is from 1992 to 2013. Its spatial resolution is 30 arc-seconds, about 1 km at the equator. However, the DMSP-OLS ceased operation in 2013, which means no data can be got ever since. So, the research period of this paper is from 1992 to 2013. The new generation NTL data of NPP-VIIRS are available from 2012 to the present day, and the spatial resolution of that is 541 m. The night light data provided by luojia-1(LJ1-01) night light remote sensing satellite (jointly produced by Wuhan University of China and some other relevant institutions) can be available from June 2018 to the present day. The spatial resolution of the LJ1-01 data is 130 m. The spatial resolution of the new generation NTL data of NPP-VIIRS and LJ1-01 is higher than that of DMSP-OLS and the data is highly real-time, but the defect of them is that the time span is short. In the future research, it is necessary to consider how to calibrate the NTL data which is got by different sensors of different institutions in different period to the same brightness reference to extract the city built-up area and extend the time series that is studied in this paper. Furthermore, our future study will consider to use the methods proposed in the newest literature [42, 43].

In addition, it deserves the attentions of government administrators and urban planners to consider how to rationally apply the results to urban planning after accurately grasping the law of urban development. Last, DMSP-OLS NTL data is an ideal spatial data source, it may become an important direction to combine the approached based on cellular automata and multi-agent to simulate and forecast urban development.

## 5. Conclusions

It is of great significance to study the laws and characteristics of spatial-temporal evolution of urban development for the purpose of determining the rational distribution of urban land and avoiding blind expansion of urban land use, and realizing the sustainable development of big cities [44, 45]. In this paper, the spatial characteristics of urban development in Beijing has been studied by using DMSP-OLS night-time light data in 1992–2013 from the following aspects: spatio-temporal scales of urban expansion, city barycenter transfer trajectory and urban development direction, space morphological changes and internal differences of urban development. A couple of conclusions can be drawn from the study:

Firstly, Beijing circularly had expanded from the center of the city to the outer space. During the whole study period, the expansion area, speed and intensity of Beijing were always the largest in the third stage from 2001 to 2007 and the least in the fourth stage from 2007 to 2013. The expansion of the city was accelerative in 2001–2007 and decelerative in 2007–2013. The expansion of the city mainly concentrated in 1996–2007, and the expanded area accounted for 92% of the total research period. The built-up area had expanded in all directions, but the expansion area to the northeast was the largest among that of all directions, and the expansion area to the north is larger than that of the south.

Secondly, the barycenter of Beijing was located in Dongcheng District in 1992 with geographical coordinates 116° 21′ 45″ E and 39° 55′ 30″ N. In 2013, the city barycenter shifted to Chaoyang District with geographical coordinates 116° 24′ 40″ E and 39° 56′ 30″. From 1992 to 2013, the barycenter of Beijing moved to east by north 28°, and the barycenter shifted from Dongcheng district to Chaoyang District with a distance of 5000.71 m.

Thirdly, during the whole study period, the urban spatial morphology of Beijing had alternated between extensive expansion and filling intensive expansion. From 1992 to 2001, the compactness index of Beijing had increased gradually, and the urban expansion type was mainly intensive filling. From 2001 to 2009, the urban compactness had decreased and the urban extension type was mainly extensive expansion. From 2009 to 2013, the compactness index had increased slightly, and the urban expansion was mainly intensive filling.

Fourth, the overall urban development in Beijing had been gradually improving, and the gap between different municipal districts had been gradually decreasing. Dongcheng District and Xicheng District as the core of the capital function regions were almost saturated in early period, and their relative development rate were the lowest. As the newly developed area, the five districts including Fangshan, Tongzhou, Shunyi, Changping and Daxing District, had the highest relative development rate and they had been at the development peak, because the government had invested much more money in the newly developed areas than that in other ones.

The result can provide scientific reference for future urban planning and development in Beijing.
